# Bio-templated fabrication of three-dimensional network activated carbons derived from mycelium pellets for supercapacitor applications

**DOI:** 10.1038/s41598-017-18895-6

**Published:** 2018-01-12

**Authors:** Junnan Hao, Yajing Huang, Chun He, Wenjun Xu, Libei Yuan, Dong Shu, Xiaona Song, Tao Meng

**Affiliations:** 1 School of Environmental Science and Engineering, Sun Yat-sen University, Guangzhou, 510275 People’s Republic of China; 2 School of Chemistry and Environment, South China Normal University, Guangzhou, 510006 People’s Republic of China; 3 Engineering Research Center of Materials and Technology for Electrochemical Energy Storage (Ministry of Education), Guangzhou, People’s Republic of China

## Abstract

In this work, a three-dimensional porous mycelium-derived activated carbon (3D-MAC) was fabricated via a facile bio-templating method using mycelium pellets as both the carbon source and the bio-template. After ZnCl_2_ activation and high-temperature carbonization, the specific thread-like chain structure of mycelium in the pellets can be maintained effectively. The hyphae and junctions of the cross-linking hyphae form nanowires and carbon nanoparticles that link with the neighboring nanoparticles to form a network structure. By adding NH_4_Cl, foreign nitrogen element doped (N-doped) 3D-MAC was obtained, which has a hierarchical porous structure composed of micropores and macropores. And the multiple pore size distribution benefits from ZnCl_2_ activation, the specific 3D structure and gas blowing. Meanwhile, the introduction of some hydrophilic groups and abundant N-containing functional groups in extrinsic N-doped 3D-MAC contributes to improving the Faradaic pseudocapacitance, respectively. A specific capacitance of 237.2 F g^−1^ at 10 mV s^−1^ was displayed, which is more than 1.5 times that of 3D-MAC. Even at the large scan rate of 500 mV s^−1^, N-doped 3D-MAC still reveals a nearly symmetric rectangular shape, demonstrating great potential as a high-performance supercapacitor electrode material due to the synergistic effects of its 3D hierarchical porous structure and various functional groups.

## Introduction

Nowadays, biological adsorption is becoming widespread for the advanced treatment of sewage that contains various heavy metal ions or spilled oil^1,2^. Mycelium pellets exhibit outstanding adsorption performance for pollutants due to its slender hyphae and the unique thread-like chain structure^3^. Detailed investigations demonstrate that the composition of mycelium pellets, unlike the cellulosic cell walls of plants, is rich in chitin which is a highly insoluble material resembling cellulose in its solubility and low chemical reactivity^4^. Therefore, our research group has prepared nano-sized hierarchical porous activated carbons (ACs) with a three-dimensional (3D) network structure through a facile bio-templating method from mycelium pellets. The templating technique has become a versatile route to the fabrication of advanced materials with controlled nano/microstructures and desired functions. Furthermore, a variety of biomass materials have been reported to prepare ACs with various microscopic morphologies, pore volume, and porosities. For instance, bamboo^5^, banana fibers^6^, corn grains^7^, coffee beans^8^, seaweed^9^, dead leaves^10^, ginkgo shells^11^, typha orientalis^12^, coconut shells^13^, catkins^14^, and pine needles^15^ have all been employed to produce different AC materials. Furthermore, some fungi have also been reported as different types of excellent bio-templates to prepare various carbon products for energy storage devices, including yeast^16^, auriculariae^17^, and mushrooms^18^. Nevertheless, almost all of these ACs obtained from the macroscopic precursors mentioned above are two-dimensional (2D) materials, which tend to suffer from poor conductivity, serious agglomeration of materials and poor electrochemical performances. Thus, compared with previous reports, this current work employs mycelium pellets with a different kind of smaller thread-like chain structure as bio-template and carbon resource to fabricate 3D hierarchical porous ACs. We demonstrate that a slender hypha in the mycelium pellets can turn into a carbon nanowire via high-temperature carbonization. Meantime, a single junction of the cross-linking hyphae would form an individual carbon nanoparticle that links with neighboring carbon nanoparticles through nanowires, leading to a unique templated 3D cross-linked structure with a conductivity and specific surface area.

Recently, supercapacitors have attracted great attention among energy storage researchers, because of their ability to provide higher energy density than the conventional electrostatic capacitors and higher power density than batteries^19,20^. Based on the charge-storage mechanism, supercapacitors can be generally classified into two categories: electrical double-layer capacitors (EDLCs) in which various carbon materials are used as electrode materials and pseudocapacitors in which certain metal oxides or conducting polymers are often used as electrode materials^21,22^. Compared with other carbon-based electrode materials, ACs have been extensively studied for EDLCs, due to their significant properties, such as high abundance, easy processability, micro-to-nano-porosity, high surface area, low cost, etc. Moreover, introducing hetero-atoms (e.g., P, S, N, and B) into ACs is a facile technique to enhance the pseudocapacitance of electrode materials^23,24^. In particular, for supercapacitors, N-doping has been claimed to increase the pseudocapacitance by acting as an electron donor to attract protons and/or strengthen redox reactions^25^.

An interesting phenomenon is that, while the mycelium pellets have a certain amount of nitrogen element, the as-prepared 3D mycelium-derived network activated carbon (3D-MAC) after high-temperature carbonization would contain only a little amount of N element^26,27^. Unfortunately, 3D-MAC achieved by direct carbonization of mycelium pellets without other N resources suffer from the small amount of N and because there are few types of N-containing functional groups. Thus, to enrich the N content and broaden the N-containing functional groups to improve the electrochemical performance, NH_4_Cl as a foreign N resource was introduced during carbonization. Herein, this work presents extrinsic N-element doped (N-doped) 3D-MAC with a 3D hierarchical porous structure, hydrophilic groups, and abundant N-containing functional groups via a facile bio-templating method with ZnCl_2_ activation and N-doping. The presence of micropores benefit from the ZnCl_2_ activation. On the other hand, there are abundant macropores, thanks to the unique 3D structure of the material and gas blowing (NH_3_ and HCl gases generated from the pyrolysis of NH_4_Cl). This hierarchical porous structure would contribute to enhancing the specific surface area and offering more transfer channels for electrolyte ions. Meanwhile, the N resource would have an effect on the N types, the wettability, and the electrochemical properties of the material as well, and these influences have been explored.

## Results and Discussion

### Characterization of samples

**Figure 1:**
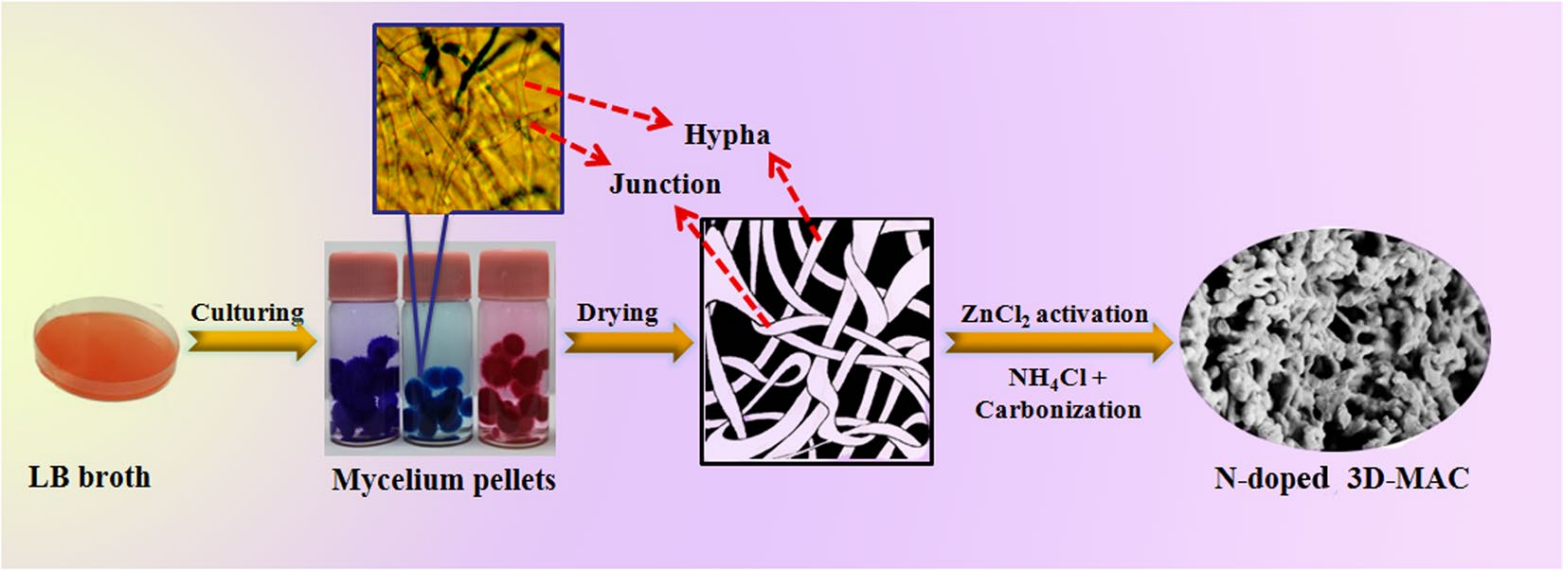
Schematic illustration of the procedure for preparing foreign N-doped 3D-MAC.

**Figure 2:**
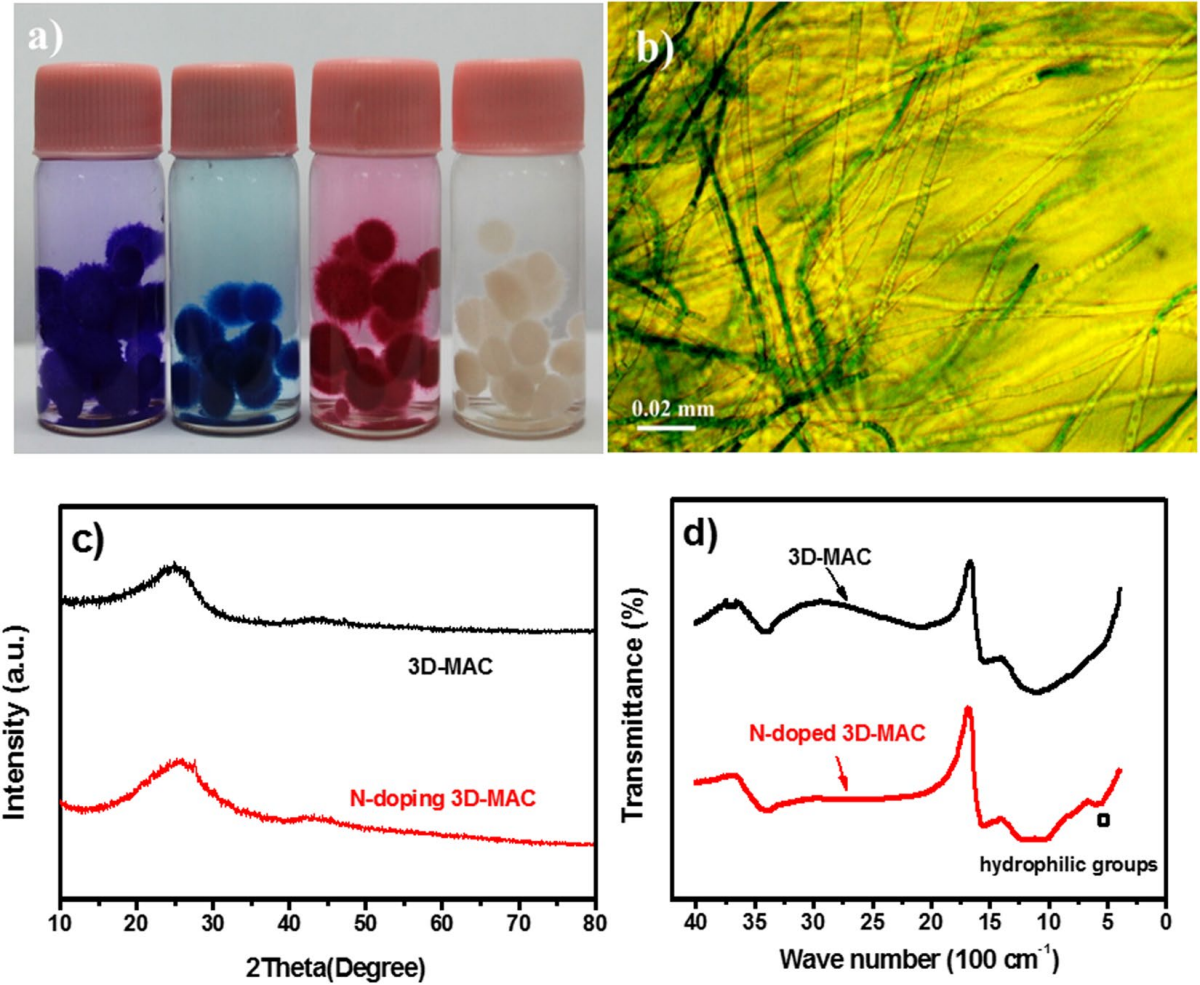
(**a**) Photograph of mycelium pellets that have absorbed different chemical dyes; (**b**) Optical microscope image of hyphae; (**c**) XRD patterns of 3D-MAC and N-doped 3D-MAC; and (**d**) FT-IR spectra of both materials.

The samples were prepared via a facile bio-templating method, as shown in Fig. 1, in which the products effectively maintain the specific thread-like chain structure of mycelium pellets via high-temperature treatment. Their unique microscopic structure gives the mycelium pellets outstanding adsorption performance, as demonstrated in Fig. 2a, in which the mycelium pellets show that they can absorb different chemical dyes, including methyl blue, methyl red, etc. Thus, the unique microscopic structure also gives the mycelium pellets the potential to be a promising bio-template and candidate for AC materials in energy storage devices. From the optical microscope images of the hyphae (Fig. 2b and Figure S1 in the Supporting Information), it can be observed clearly that they are extremely slender. Under an external force, these slender hyphae easily bend, cross-link, and then form mycelium pellets that feature abundant nodes of hyphae and a specific thread-like chain structure. This thread-like chain structure facilitates connections with the neighboring carbon nanoparticles (derived from hyphae nodes) as well. Thus, the unique microscopic structure is conducive to producing novel AC materials with a 3D cross-linked network structure after high-temperature carbonization.

The XRD patterns of the 3D-MAC and N-doped 3D-MAC samples are presented in Fig. 2c. Both of them reveal the absence of sharp and strong peaks, indicating the amorphous state of two samples. The broad peaks that appear at 2*θ* values of approximately 24° and 44° in the XRD pattern of 3D-MAC correspond to the (002) and (100) planes of the graphitic lattice respectively, which indicates graphitic structures are formed during the heat treatment^28^. Furthermore, the pattern of the N-doped 3D-MAC reveals similar XRD results to the 3D-MAC, demonstrating that the introduction of extrinsic N element does not significantly change the crystalline structure of 3D-MAC.

3D-MAC and N-doped 3D-MAC were investigated by Fourier transform infrared (FT-IR) spectroscopy (Fig. 2d). The observed bands in both samples at 3400–3100 cm^−1^ correspond to stretching vibrations of O-H in hydroxyl groups. The bands at 1560–1578 cm^−1^ are attributed to C=O stretching vibrations^29^. The bands at 1100–1391 cm^−1^ are ascribed to C-O stretching vibrations in alcohols, phenols, acids, esters, and ethers^30^. A weak band (520–600 cm^−1^) in the N-doped 3D-MAC spectrum reveals the presence of some hydrophilic functional groups after N-enrichment, which increases the surface wettability of the materials. These functional groups are also confirmed by the X-ray photoelectron spectroscopy (XPS) data^31^. Thus, compared with the ordinary 3D-MAC, N-doped 3D-MAC have variation variety of foreign N types and hydrophilic Cl-containing functional groups which can improve the contact between the electrolyte and electrode material, and modify the electron donor/acceptor properties^32^. Figure S2 contains the XPS analytical results for the 3D-MAC and N-doped 3D-MAC materials. There are four peaks appearing at 198.0, 284.7, 399.5, and 532.5 eV in N-doped 3D-MAC, which correspond to Cl 2p, C 1 s, N 1 s, and O 1 s, respectively, demonstrating the presence of Cl and N elements in this sample^33^. The presence of N makes the mycelium-derived 3D-MAC different from the ACs derived from other types of biomass, including waste paper, cotton, etc^34,35^. The XPS analysis also reveals that there is 3.3 at% and 7.7 at% N in 3D-MAC and N-doped 3D-MAC, respectively. The extra N element would be introduced into the 3D-MAC structure when the NHCl_4_ is introduced during the heating process. Additionally, Figure S2b indicates that there are four different types of N-containing functional groups in N-doped 3D-MAC, which correspond to pyrrolic N (398.3 eV), pyridinic N (400.1 eV), graphitic N (401.5 eV,) and oxidized N (403.4 eV)^22^. The N 1 s peak in 3D-MAC (Figure S2c), however, can be mainly resolved into two components, which are centered at approximately 398.3 and 400.1 eV, corresponding to the pyrrolic N and pyridinic N, respectively. The XPS analysis also reveals the different N functionalities in 3D-MAC and N-doped 3D-MAC, as shown in Table 1. It is clear that, after N-enrichment, there are more extrinsic N-containing functional groups linked to oxygen in N-doped 3D-MAC than that in the 3D-MAC, which is consistent with the FT-IR patterns as well.

**Table 1:** Proportions of N types in XPS N 1 s analysis.

N functionalities	pyrrolic N	pyridinic N	graphitic N	oxidized N	total content
N-doped 3D-MAC	46.75%	33.46%	18.43%	1.37%	7.7 at%
3D-MAC	69.95%	30.05%	0%	0%	3.3 at%

**Figure 3:**
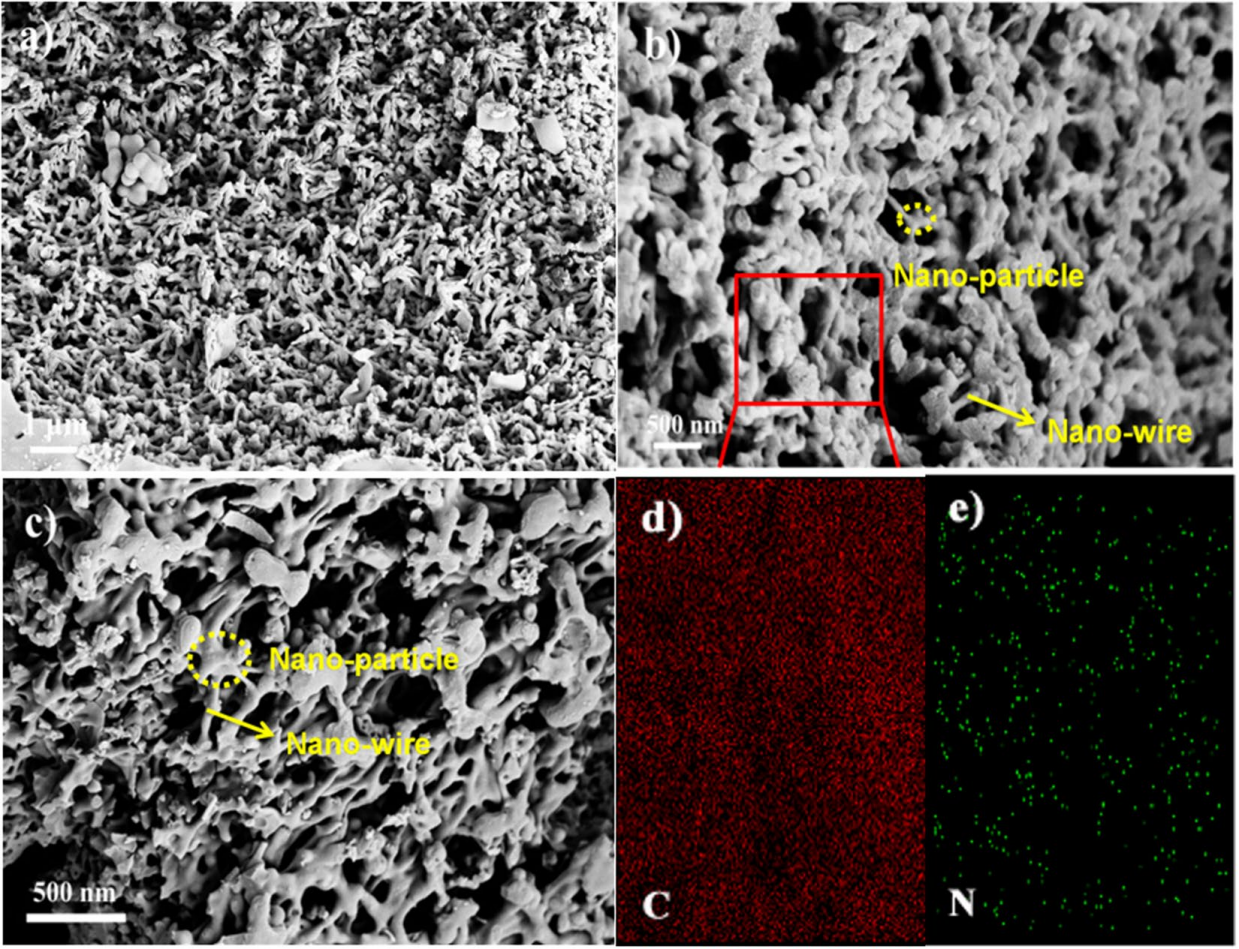
SEM images: (**a**) N-doped 3D-MAC; (**b**) higher magnification of N-doped 3D-MAC; (**c**) 3D-MAC; Mapping analysis of N-doped 3D-MAC: (**d**) C element and (**e**) N element.

The surface morphology of N-doped 3D-MAC was characterized by scanning electron microscopy (SEM) (Fig. 3). Figure 3a,b indicates that N-doped 3D-MAC has a 3D continuously cross-linked structure, vividly maintaining the specific thread-like chain structure of the bio-template, which is evidently different from the cases of the ACs with a 2D structure derived from dead leaves that were reported by Biswal *et al*.^10^ and the fungi reported by Zhu and co-workers^36^. From Fig. 3b, it is clear that a slender hypha in mycelium pellets is turned into a carbon nanowire via carbonization treatment, and that large numbers of carbon nanoparticles are stitched together by them. Apparently, a single junction of the cross-linking hyphae forms an individual carbon nanoparticle after carbonization treatment, which is linked with neighboring carbon nanoparticles through the carbonized hyphae, resulting in a unique templated 3D cross-linked structure. Such a cross-linked 3D structure effectively provides more channels in the material, which facilitates the entrance of electrolyte ions and/or electrons into the interspace voids, strongly contributing to enhancement of the electrochemical performance. In addition, abundant pores, resulting from the 3D cross-linked structure rather than ZnCl_2_ activation, with sizes ranging from dozens of nanometers to several micrometers can be observed. The surface morphology of 3D-MAC is shown in Fig. 3c. Compared with that of the N-doped 3D-MAC under the same magnification, the 3D-MAC also exhibit a similar 3D cross-linked structure except that the nanowires are thicker and the nanoparticles are larger. The main reason may be that the NH_3_ and HCl gases derived from the thermal decomposition of NH_4_Cl during carbonation would help to disperse the carbon precursor in the N-doped 3D-MAC sample. From the mapping analysis (Fig. 3d,e), it is clearly observed that the N and C elements are uniformly distributed in the 3D carbon material. The introduction of a certain amount of N-containing functional groups in the N-doped 3D-MAC sample contributes to the enhancement of the Faradaic pseudocapacitance^37^.

**Figure 4:**
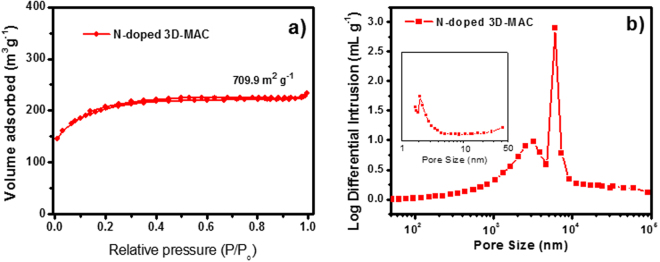
(**a**) Nitrogen sorption isotherms; and (**b**) Pore size distributions of both samples, with the inset an enlargement for the smallest pore sizes.

The nitrogen adsorption-desorption isotherms of 3D-MAC and N-doped 3D-MAC are presented in Fig. 4a. The curves of both samples, based on the International Union of Pure and Applied Chemistry (IUPAC) classification, have shapes consistent with the I-type, which makes them similar to the adsorption-desorption isotherms of ACs produced from coffee beans, as reported by Rufford and his co-workers^8^. Additionally, the specific surface area of electrode materials is a significant parameter, as it affects a variety of characteristics, for instance, the specific capacitance^34^. On the basis of the N_2_ adsorption, the specific surface areas of 3D-MAC and N-doped 3D-MAC are determined to be 586.3 and 709.9 m^2^ g^−1^, respectively. This value for N-doped 3D-MAC is higher than that of ACs fabricated from biomass, such as waste paper activated by KOH (525.0 m^2^ g^−1^) and Eichhornia crassipes (common water hyacinth) activated by ZnCl_2_ (579.9 m^2^ g^−1^)^34,38^. Notably, there was significant adsorption in the low-pressure region, which confirmed the presence of micropores^39^. Whereas, no conspicuous hysteresis loop could be observed in either sample, revealing that there were fewer mesopores. More direct evidence for the formation of macropores was provided by mercury intrusion analysis (Fig. 4b). It is clearly revealed from Fig. 4b that both as-prepared samples possess abundant micropores (<2 nm). An abundance of micropores may be promoted by the activating agent (ZnCl_2_). During the activation process, ZnCl_2_ works as a dehydration agent during carbonization. This result leads to the charring and aromatization of the carbon skeleton, leaving the abundant micropore structure^27^. Compared with 3D-MAC, however, the N-doped 3D-MAC have more macropores (>50 nm). The macropores may have resulted from the 3D cross-linked structure and gas blowing, which can be confirmed by the SEM images as well. Such a hierarchically porous structure would be beneficial to contact of the electrolyte with the electrode materials and electrolyte ion diffusion, thus leading to outstanding performance as electrode materials for energy storage devices^32^.

### Electrochemical properties

**Figure 5:**
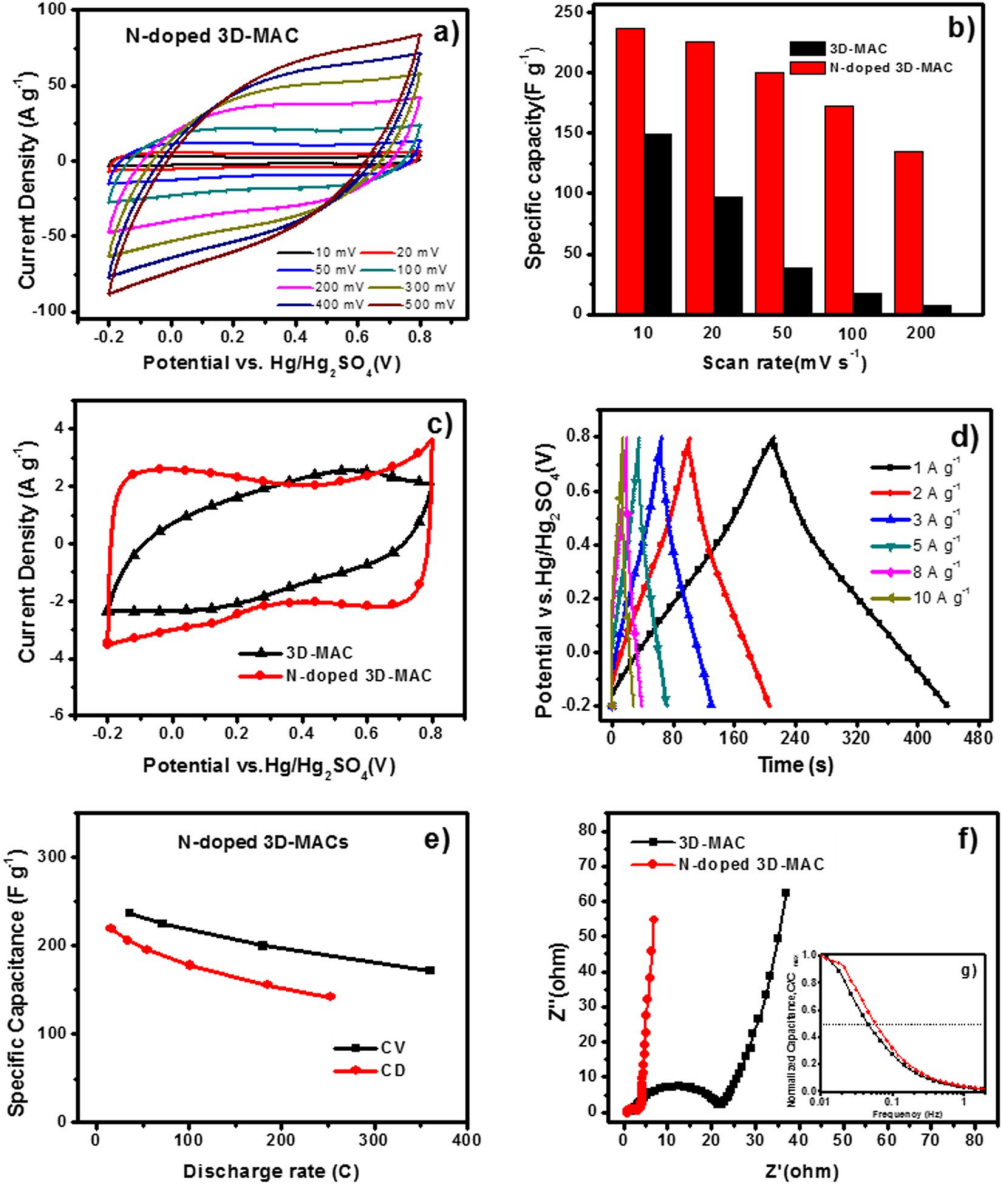
(**a**) CV curves of N-doped 3D-MAC at various scan rates; (**b**) Specific capacitance values of both samples; (**c**) CV curves of both samples at 10 mV s^−1^; (**d**) Galvanostatic CD curves of N-doped 3D-MAC at different current densities; (**e**) the corresponding specific capacitance of N-doped 3D-MAC electrode as a function of the discharge rate; (**f**) Nyquist plots of both electrodes, with the inset showing their frequency response.

The electrochemical performances of 3D-MAC and N-doped 3D-MAC were investigated in 1.5 mol L^−1^ H_2_SO_4_ aqueous solution. Cyclic voltammograms (CVs) for the as-prepared samples are shown in Fig. 5a and Figure S3a. The N-doped 3D-MAC shows a nearly quasi-rectangular CV curve, even at 500 mV s^−1^ (Fig. 5a), revealing its low contact resistance and excellent capacitive behavior at high current loads, which is mainly due to the presence of hydrophilic groups and N-containing functional groups (especially graphitic and oxidized N and pyridinic N)^37,40^. Whereas, the quasi-rectangular curves of the conventional 3D-MAC electrode only can be observed at 10 and 20 mV s^−1^ (Figure S3a). The curve shape is severely distorted if the scan rate climbs to 100 or 200 mV s^−1^, because of the poor rate performance and significant internal resistance of this electrode. Moreover, based on Eq. (1) in supporting information, the specific capacitance of N-doped 3D-MAC is calculated to be 237.2, 225.3, 200.3, 172.2, 134.9, 110.2, 92.4, and 79.0 F g^−1^ at 10, 20, 50, 100, 200, 300, 400, and 500 mV s^−1^, respectively; in contrast, for the ordinary 3D-MAC, the specific capacitances are 149.5, 97.0, 38.0, 17.0 and 6.9 F g^−1^ at 10, 20, 50, 100, and 200 mV s^−1^, respectively (as shown in Fig. 5b).

To obtain more information on the various electrochemical features of the N-doped 3D-MAC and 3D-MAC samples, the CV curves were compared at 10 mV s^−1^, as shown in Fig. 5c. It is clear that the CV curve of N-doped 3D-MAC shows a nearly symmetrical rectangular shape with a larger encircling area, which suggests excellent supercapacitive behavior. A weak and broad peak exists in the curve, however, possibly resulting from the introduction of N-containing functional groups (especially oxidized N)^40^. In contrast, the CV curve of 3D-MAC appears relatively smaller and less quasi-rectangular under the same testing conditions, demonstrating its poor capacitance behavior. Consequently, the adequate N content and hydrophilic functional groups in microbial carbons play vital roles in enhancing the electrochemical capacitive behavior. Specifically, the hydrophilic N-containing functional groups, such as oxidized N-linked to oxygen, would contribute to the diffusion of electrolyte ions (H^+^) and modify the electron donor/acceptor properties and Faradaic pseudocapacitance^12^. Furthermore, as is well-known, the CV loop shape of supercapacitors tends to be rectangular when the contact resistance of the electrode is small. Additionally, larger resistance distorts the loop of CV curves, leading to a narrower circle with an oblique angle^41^.

Figure 5d contains galvanostatic charge-discharge (CD) curves. The curves of N-doped 3D-MAC reveal the typical triangular shape, which indicates outstanding capacitive properties. Furthermore, the current-resistance (IR) drops in N-doped 3D-MAC curves are relatively small, which may be mainly ascribed to the unique 3D hierarchically porous structure and the hydrophilic functional groups, including graphitic N and oxidized N. The unique 3D porous structure and hydrophilic N-functional groups would contribute to the diffusion of the electrolyte ions (H^+^) as well as the wettability on the surface of the electrode^32^. The IR drops in the curves of 3D-MAC (Figure S3b) under the same current densities are larger, however, which may be primarily produced by large internal resistance. Under large scanning rates, these IR drops may also distort the shape of CV curves, which is in agreement with the CV data previously presented as well.

A linear-relationship, according to Eq. (2) in supporting information, between the specific capacitance and the discharge current density was studied (Figure S3c). Notably, N-doped 3D-MAC shows its maximum specific capacitance of 219.4 F·g^−1^ at 1 A g^-^, which is superior to the performance of advanced carbons (less than 200.0 F g^−1^ in aqueous electrolyte)^42^, including heteroatom-enriched ACs in concentrated sulfuric acid (approximately 190.0 F g^−1^ at 1.0 A g^−1^ in H_2_SO_4_ electrolyte)^43^, ACs derived from rice husks (about 200.0 F g^−1^ at a constant current of 0.2 mA)^44^, and AC samples fabricated from fungi (nearly 196.0 F g^−1^ in 6.0 M KOH aqueous electrolyte)^36^. As the discharge current density increased to 10 A g^−1^, the decay rate of the specific capacitance was approximately 34.4%. Whereas, the specific capacitance of 3D-MAC is lower, 154.5 F g^−1^ at 1 A g^−1^ for example, which may be due to its massive internal resistance^45^. Only about 2.6% of the capacity was maintained when the discharge density rose to 10 A g^−1^. In the CD process for 3D-MAC, resulting from the reduced transferability, there was not sufficient time for electrolyte ions to penetrate the electrode, and CD could not ultimately proceed, leading to low utilization of active material. Hence, the specific capacitance decreased dramatically.

To explore the relationship between the specific capacitance values of N-doped 3D-MAC calculated from the CV tests and those estimated from the galvanostatic CD experiments, the discharge times in the CV tests (discharge times at 10, 20, 50 and 100 mV s^−1^, respectively) and the galvanostatic CD experiments (those at 1, 2, 3, 5 and 10 A g^−1^) were converted into the discharge rate values. Figure 5e presents the specific capacitance values, which were calculated from both test methods, versus the discharge rates. When the discharge rate is low, the specific capacitance values obtained from the CV measurements are slightly higher than those calculated from the galvanostatic CD methods at the same discharge rate. As the discharge rate climbs, both types of values reveal a similar decreasing tendency. Nevertheless, all the results indicated that the specific capacitance values obtained by both the CD and the CV methods at a similar discharge rate are comparable^43^.

The cycling stability of the N-doped 3D-MAC and 3D-MAC electrodes was further evaluated via repeating the CV experiments at 20 mV s^−1^ for 5,000 cycles (Figure S3d). After 5,000 cycles, the capacity retention of N-doped 3D-MAC is more than 99.6%, indicating great cycling performance for supercapacitors. The outstanding performance is mainly ascribed to the presence of hydrophilic functional groups and hierarchical porous structure, which facilitates the movement of electrolyte ions into/out of the internal pores of the material during the charge and discharge process. Whereas, the decay rate of the 3D-MAC electrode reached 36.5% after 5,000 cycles.

Electrochemical impedance spectroscopy (EIS) measurements were conducted to obtain in-depth insight into the capacitive and resistive properties of the N-doped 3D-MAC and 3D-MAC samples (Fig. 5f). From an overall perspective, the Nyquist plots are composed of a line in the low-frequency region, which corresponds to the diffusive resistance of the electrolyte ions in the pores of the electrode, along with the diffusion of electrons in the host material, and a semicircle in the high-frequency region, which tends to be related to the electrochemical reaction process in the electrode material^46^. Moreover, the diameter of the semicircle closely represents the charge-transfer resistance (*R*_ct_), which is associated with the reversibility of the electrochemical reactions^47^. By calculation, the *R*_ct_ values of the N-doped 3D-MAC and the ordinary 3D-MAC are approximately 6 Ω and 22 Ω, respectively. The *R*_ct_ of N-doped 3D-MAC is lower than that of the ordinary 3D-MAC. The larger *R*_ct_ of 3D-MAC distorts the loop of the CV curve, which is also consistent with the CV data discussed in the previous section. When comparing the frequencies at which capacitance drops to 50% of its maximum value (*f*_max_, Fig. 5g inset), based on Eq. (3), we clearly see that the N-doped 3D-MAC also demonstrates a better frequency response (~0.058) than that of 3D-MAC (~0.043), which likely due to hydrophilic N-containing functional groups and the hierarchically porous structure of this sample (Fig. 4b)^48^.

## Conclusions

In summary, mycelium pellets with their particular thread-like chain structure were employed as the bio-template and carbon source to fabricate 3D cross-linked 3D-MAC for supercapacitor applications. By adding a foreign N source, N-rich 3D-MAC featuring abundant N-containing functional groups, including pyrrolic N, pyridinic N, oxidized N, and graphitic N, was achieved. Owning to ZnCl_2_ activation, its 3D cross-linked structure, and gas blowing, the N-doped 3D-MAC have a unique hierarchical porous structure. Benefiting from the 3D hierarchical porous structure and hydrophilic functional groups, the N-doped 3D-MAC exhibit better electrochemical performance than the conventional 3D-MAC. At 1 A g^−1^, the N-doped 3D-MAC displays a specific capacitance of 219.4 F g^−1^, which is approximately 1.5 times that of 3D-MAC under the same test conditions. After 5,000 cycles, more than 99.6% of the capacitance is still preserved, which reveals its attractive cycling stability. This excellent performance makes N-doping 3D-MAC promising candidates as electrode materials for supercapacitor applications.

### Experimental section

#### Cultivation and collection of mycelium pellets

There are main three stages in the formation of the mycelium pellets which acted as bio-template and carbon source: mold condensation, germination, and the growth of mycelium, respectively. Luria-Bertani (LB) broth was selected as the nutrient solution, which provided nutrients and a suitable place for mold growth. The agitation rate and temperature were kept at 150 rpm and 30 °C for 72 h. After 7 days, the mold could sprout and grow slender mycelia. Subsequently, mycelium pellets were formed under external force. The cultured mycelium pellets, as precursors for 3D-MAC, were soaked in deionized water for 0.5 h, and then washed several times to remove the remaining LB broth.

#### Preparation of 3D-MAC and N-doped 3D-MAC materials

AC samples were fabricated as follows. First, 2 g mycelium pellets was activated using 200 mL ZnCl_2_ aqueous solution (1.5 mol L^−1^) under stirring for 24 h at ambient temperature. Subsequently, the samples were filtrated and then dried in a vacuum-freezing drying oven at 70 °C. Then, the precursors were carbonized starting from ambient temperature at a designed heating rate (i.e., 2 °C min^−1^ as recommended) up to 750 °C for 2 h in a tube furnace under an N_2_ atmosphere (SK-G08123K, 120 cm in length by 10 cm in diameter) with NH_4_Cl and without NH_4_Cl, respectively. The final samples, which were washed in hot deionized water several times to remove the residual ZnCl_2_, were denoted as N-doped 3D-MAC (as shown in Fig. 1) and 3D-MAC, respectively^49^.

## Electronic supplementary material

Supplementary Information
