# Supplementary material for: Bio-templated fabrication of three-dimensional network activated carbons derived from mycelium pellets for supercapacitor applications
Source: Sci Rep. 2018 Jan 12;8:562. doi: 10.1038/s41598-017-18895-6 (PMC5766499; doi:10.1038/s41598-017-18895-6)
Supplement: Supplementary file 1 — Supplementary Information [file 41598_2017_18895_MOESM1_ESM.pdf]

Bio-templated fabrication of three-dimensional network activated carbons derived from mycelium pellets for supercapacitor applications

Junnan Hao [a], Yajing Huang [a], Chun He [a*], Wenjun Xu [a], Libei Yuan [b], Dong Shu [b,c*], Xiaona Song [b], Tao Meng [b]

[a]*School of Environmental Science and Engineering, Sun Yat-sen University, Guangzhou 510275, People's Republic of China*

[b]*School of Chemistry and Environment, South China Normal University, Guangzhou 510006, People's Republic of China*

[c]*Engineering Research Center of Materials and Technology for Electrochemical Energy Storage (Ministry of Education)*

**Chun He, Email address: hechun@mail.sysu.edu.cn (C. He); Tel.: +86 20 39332690.*

**Dong Shu, Email address: dshu@scnu.edu.cn.*

Material characterization

The powder X-ray diffraction (XRD) technique was employed to characterize the crystallographic structures of the N-doped 3D-MAC and 3D-MAC powders, the data were collected on an X-ray generator (D/MAX 2200 VPC) with Cu K α radiation at a specific step of 0.3 ^{o} s^{-1}. The surface functional groups and morphology of samples were characterized using the Fourier transform infrared spectrometry (FT-IR, EQUINOX 55-type), the scanning electron microscope (SEM, ZEISS Ultra55) and X-ray photoelectronspectroscopy (XPS, ESCALAB250) measurements. The Brunauer-Emmett-Teller (BET, ASAP2020) method, based on the adsorption-desorption of N_2, was adopted to analyze the specific surface area

and micro-and mesoporous distribution of the samples. The distribution of macropore was analyzed using mercury intrusion analysis.

Electrochemical measurements

The CHI660E electrochemical workstation (Shanghai CH Instruments Co., China) was used to carry out the electrochemical measurements through a three-electrode system, in where the reference electrode (Hg/Hg_2SO_4),the working electrode prepared by mixing active materials, polyvinylidene fluoride and acetylene black (8:1:1), as well as auxiliary electrode (Pt) existed. Cyclic voltammograms (CVs) data were obtained from -0.2 to 0.8 V at the scan rates of 10 ~ 500 mV s^{-1}. Galvanostatic charge-discharge (CD) data were recorded from -0.2 to 0.8 V at different current densities ranging of 1 ~ 10 A g^{-1}. Cycle performance experiments were conducted under the scan rate of 20 mV s^{-1}.

According to the following Eqs. (1) and (2), CVs and galvanostatic CD data were employed to calculate the specific capacitances (C, F g^{-1}) of electrodes respectively:

$$C_{(CV)} = Q/(\Delta V \times m) \qquad (1)$$

$$C_{(CD)} = (I \times \Delta t)/(m \times \Delta V) \qquad (2)$$

Where C represents the specific capacitance (F g^{-1}), Q is the cathodic charge (C), ΔV is the potential window width (V), and m is the mass of active material (g).

Electrochemical impedance spectroscopy (EIS) measurements were tested during the frequency range of 0.01 ~ 1×10^5 Hz, the open circuit potential of 0.3 V was applied to the electrodes with an alternating current amplitude of 5 mV. The gravimetric capacitance, C (F g^{-1}), was calculated according to

$$\mathrm{C} = 2|\mathrm{Im(Z)}|/2\pi f\left[\mathrm{Im}(Z)\times\mathrm{Im}(Z)+\mathrm{Re(Z)}\times\mathrm{Re(Z)}\right]\mathrm{m} \qquad (3)$$

Where f represents the operating frequency (Hz), Re(Z) and Im(Z) are the real and imaginary parts of the total device resistance (Ohm) and m is the mass of active material (g)

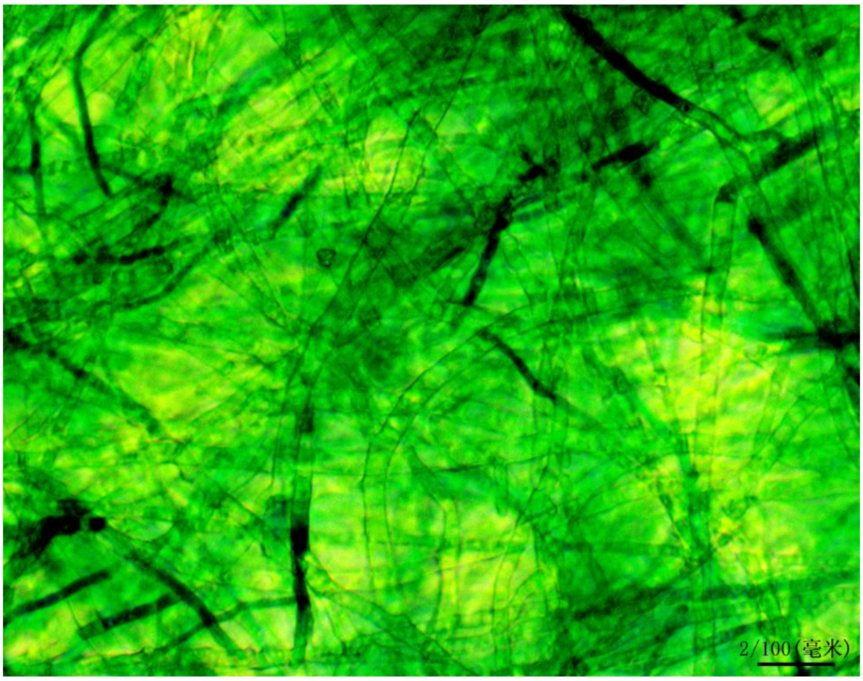

Figure S1 Optical microscope result of hyphae

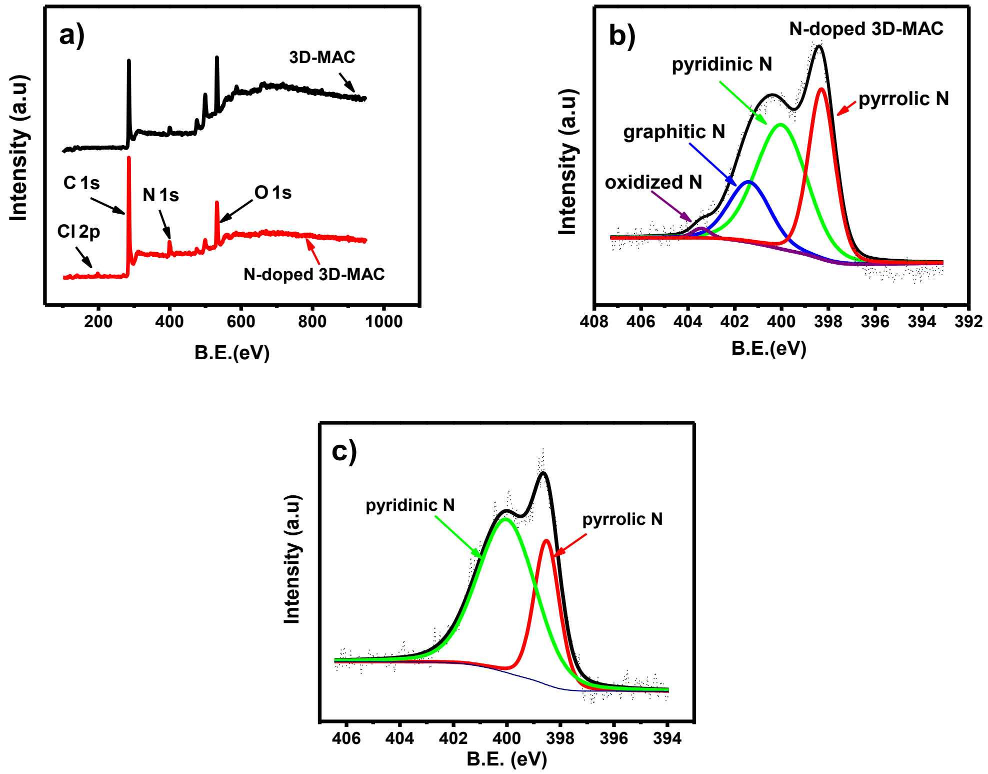

Figure S2 a) XPS survey scan of both materials; N 1s spectrum of b) the N-doped 3D-MAC and c) the 3D-MAC.

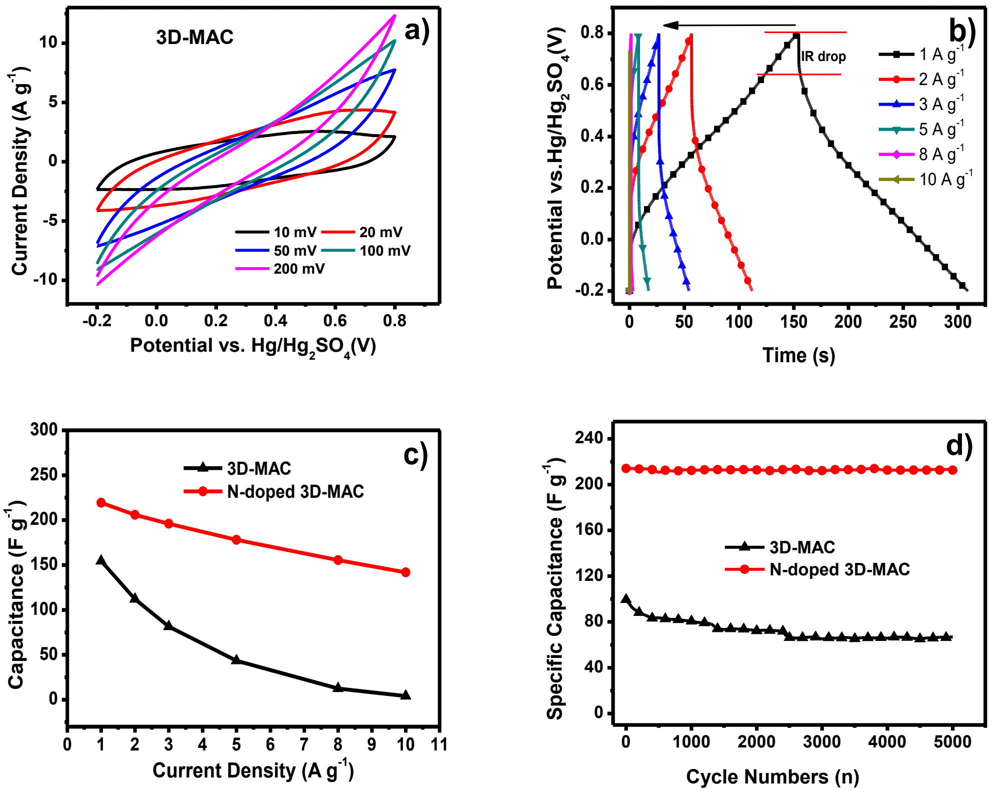

Figure S3 a) CV curves of 3D-MAC at various scan rates; b) Galvanostatic CD curves of 3D-MAC at different current densities; c) the rate performances of both electrodes and d) Cycle performances of both electrodes.
